# In-room Voice-Based AI Digital Assistants Transforming On-Site Hotel Services and Guests’ Experiences

**DOI:** 10.1007/978-3-030-65785-7_3

**Published:** 2020-11-28

**Authors:** Dimitrios Buhalis, Iuliia Moldavska

**Affiliations:** 1 Department for Informatics, Technical University of Munich, Garching bei München, Bayern Germany; 2 Smart Tourism Education Platform (STEP) College of Hotel and Tourism Management, Kyung Hee University, Seoul, Korea (Republic of); 3 Department of Tourism and Service Management, MODUL University Vienna, Vienna, Wien Austria; Bournemouth University Business School, Bournemouth, BH12 5BB UK

**Keywords:** Voice-based digital assistants, Artificial intelligence, Internet of Things, Human-computer interactions

## Abstract

Voice-based artificial intelligence (AI) devices transform human-computer bidirectional interactions with new touchpoints. Despite the recent release of purpose-developed in-room voice assistants for hotels, they have not been widely deployed by hospitality companies. There is limited research on the phenomenon of voice-based digital assistants and a research gap in their adoption by hotels for automating workflows and enhancing guests’ experiences. This study analysed the role of voice devices for mediating interactions between hotels and guests from both the hospitality technology providers’ and guests’ perspectives. This was done by the means of inductive qualitative research using 28 semi-structured interviews. The findings revealed that benefits associated with the application of voice-based digital assistants in hospitality outweigh the drawbacks for both hotels and guests. The paper proposes a model which illustrates the essence of speech-based interactions between hotels and guests via voice assistants. This concept contributes to human-computer interactions in the hotel industry.

## Introduction

Technology, smartness, robotics, Artificial Intelligence (AI) revolutionise tourism and hospitality industries, by reengineering the entire ecosystem [8–10]. Intelligent automation represented by both embodied and disembodied AI is likely to disrupt most of hotel operations, as safety remains the main value of all COVID-19 era travels [53]. What was regarded as a disadvantage of automation [43], the loss of human touch in interactions, is now considered as an advantage [19, 23, 44]. AI and voice recognition technology are integral parts of the so-called ‘new normal’ hospitality [10, 30]. In fact, 78% of hospitality companies are expecting voice-activated devices becoming mainstream for room lights and temperature controls [35]. The technology itself can bring new challenges, as once widely adopted, it may reveal its technical imperfection causing customers’ dissatisfaction. This paper aims to examine the role of in-room AI-empowered voice-based digital assistants in enhancing hotel services and experiences, the benefits and limitations linked to the introduction of voice-activated devices for operating hotel businesses as well as mediating interactions with guests.

## Literature Review

Voice recognition technology has been a popular research topic covered by many scholars in the last decade [5, 18, 21, 27, 41]. However, such studies are often focused on the implications of the technology in private households rather than hotel spaces. The existing literature [12, 25, 31, 36, 38, 40] on AI and automation in hospitality prioritise more established and widely accepted technologies and does not investigate voice assistants in detail. Studies dedicated to the adoption of voice-based assistants by hotels are very limited [15, 17]. Due to the overall low level of adoption of the technology by hotels they is insufficient for building a theoretical framework.

### Voice-Based Digital Assistants: Main Definitions

Voice recognition technology has been around for a while. However, major developments have only emerged with the launch of *Apple Siri* in 2010, *Microsoft Cortana* in 2013, *Amazon Echo* in 2014 and *Google Assistant* in 2016. Interchangeable terms are used for voice-based virtual service robots [45], including but not limited to, ‘AI voice assistants’ [21, 41], ‘intelligent virtual assistants’ [39], ‘voice-based digital assistants’ [37], ‘virtual voice assistants’ [27], ‘intelligent personal assistants’ [22] and ‘digital voice assistants’ [26]. Though scholars do not always agree on the terminology, most describe a technology that uses voice input to process information and reply with relevant actions. A voice-based assistant can be a software integrated in a smartphone or computer, like *Apple Siri*, or exist in the form of a standalone device, e.g. *Amazon Echo*, *Alibaba Tmall Genie*, *Apple HomePod*, *Google Home* [37].

Modern voice-activated devices consist of conversational AI that allows people to communicate with machines in the same way they would with other people. Typically, conversational AI includes Automatic Speech Recognition (ASR), Natural Language Processing (NLP) and Text-to-Speech (TTS). ASR takes the audio stream, transcribes it into text and then passes to the NLP and its components for analysis [1]. As for the NLP, it looks for the meanings of voice inputs in a certain context, using the knowledge about human natural language [13]. Then, TTS automatically converts a text into a synthesised speech, based on broad vocabulary options [33].

### Implications of Voice Assistants for Hospitality

Many hoteliers regard AI-empowered voice solutions as a top impacting technology [23]. This explains the growing number of experiments with voice-based digital assistants, launched to enhance hotel services and streamline guests’ experiences [14]. In 2016, *Aloft* installed *Apple Siri*’s in-room tablets, whereas *Wynn Resorts Las Vegas* equipped 4000 rooms with *Amazon Echo* speakers [20]. Following the example of these pioneers, voice technology was later introduced in many other properties. In 2018, *InterContinental Hotels Group* partnered with *Baidu* to use customised devices in China [44]. In 2018, *Amazon* launched *Alexa for Hospitality*, developed as a room hub for a guest-centric experience [2]. *Marriott International* became the first partner of the product and *The Charlotte Marriott City Centre* in North Carolina was the first location where the novel device had been implemented [11]. This particular kind of AI enabled voice-activated assistant offers a hotel-specific functionality and addresses the main concerns surrounding voice-activated devices, their security, as it is set to delete recordings automatically every 24 h. Some hoteliers, however, are still doubting *Amazon*’s interest in solely selling affordable devices [37]. Thus, to reassure guests, they reset devices manually daily or delegate the integration with *Amazon* to technology providers, that intermediate the integrations of speakers into hotels’ systems. Some other hoteliers refer to new voice assistants with advanced security features, which have been recently developed around the world, such as *Aragon* [38], *Angie Hospitality* [3], *Houndify* [42].

### Human-Computer Speech-Based Interactions

Consumers increasingly rely on search engines when looking for hospitality products before arrival and during their stays [37]. The idea of a technologically empowered voice mediation between consumers and brands made companies reconsider the new marketing role of voice. Any human-computer interaction has core features of human’s input and computer’s output. Voice-based assistants have modified such interactions with new voice touchpoints [32]. The absence of physical embodiment stimulates more personal interactions, replacing missing visual touchpoints with voice [41]. AI voice bots offer people a pleasant and convenient experience since they do not disagree and do not overload their outputs with more than one suggestion at a time [28]. A voice-based assistant is a purely consumer-centric technology, as it does not work without humans’ input. This makes customers participate in the service delivery process, co-creating their own experiences [34]. Voice-based assistants help people not only to get unusual virtual experiences, but also to customise physical ones, while the conversational AI collects data from each interaction for further AI analysis.

Voice identity can enhance logos and slogans as a part of branding [28]. Thus, businesses can prioritise VEO (Voice Engine Optimisation) strategies, over those of SEO (Search Engine Optimisation), in their ongoing digital marketing activities [44]. The concept of voice being an interface of the digital ecosystem inspired voice assistants’ producers to introduce 3rd party integration opportunities. Brands can create *Skills* for *Amazon Alexa* devices and *Actions* for those of *Google Home* to offer consumers a seamless way of accessing services or products via their personal devices. From the commercial point of view, *Skills* or *Actions* could be an efficient distribution channel. From the marketing perspective, they improve brands’ context-awareness as many customers use voice to navigate the web and can customise the experience.

## Methodology

As voice technology in hotels is relevantly new and there is insufficient literature on the field, exploratory research was adopted to develop theory and identify variables for conducting quantitative research in the future. A qualitative approach was selected [16] because it helps investigate all aspects and how people engage with this technology. This was essential for examining the role of voice-based devices in hospitality as it is largely an unexplored area. Following existing health and safety regulations during the COVID-19 lockdown, online in-depth semi-structured interviews were selected. This proved to be efficient when it is impossible to personally observe a phenomenon or meet interviewees in person. Pre-defined open-ended questions were created based on the literature findings and used in this study as guidelines rather than a script:

1. How can you describe the current role of digital voice devices in hotel services?
2. Which service tasks in hotels, in your opinion, can be automated with voice devices?
3. What are the advantages of using digital voice assistants for a hotel?
4. What are the barriers for the adoption of voice-activated devices by a hotel?
5. How can hotel guests benefit from having a smart speaker in their rooms?
6. Which functions of digital voice devices are most useful for hotel guests?
7. What is your opinion as to why some guests resist using smart speakers in rooms?
8. Speaking about the current COVID-19 pandemic, in what way may it affect guests’ attitude to face-to-face service delivery?
9. How do you see the future of in-room digital voice assistants in hotels?

The research benefited from 7 interviews with hospitality technology providers, that had introduced voice devices in hotels and had valuable insights as well as feedback to share. Consumers were represented by 21 millennial users [29], born between 1981 and 1994, who have been using voice-based assistants for personal purposes. The consumers’ age range was applied to interview people that would feel more comfortable to use and take opportunities new technologies offer. The list of technology companies was identified using purposeful or purposive sampling [46] through secondary data sources; particularly online articles about the examples of voice speakers’ use in hotels. Hence, 78 companies with relevant expertise were selected and contacted via email, 2 of which were additionally contacted during *VOICE Global Summit*. 2 more companies were contacted using references from existing participants, making a sample partially created by the means of snowballing method [46]. Out of all contacted companies, 11 (13.75%) replies were received, and 7 (8.75%) interviews were conducted. The consumer sample was created using a snowballing method. A Facebook post invited experienced users of voice assistants. Their expectations and perceptions of hotel service delivery through smart speakers was examined. All participants were selected without any geographical limitations, but with the focus on matching experiences. Out of 27 consumers contacted between, 23 (85.19%) responded, and 21 (77.78%) interviews were conducted in June and July 2020. Overall, 28 interviews were conducted for this research, and the point of saturation was reached after the 23rd interview.

All arrangements and communication with participants were done online. There is an obvious advantage to online interviews, as they provide an opportunity for a researcher to reach participants in diverse locations worldwide. The interviewees’ locations breakdown was as follows: UK (12), US (3), Netherlands (3), Ukraine (3), UAE (2), France (1), Germany (1), Spain (1), Poland (1), Bermuda (1). Interviews were conducted via Zoom (50%) and Email (50%), using the tools most convenient for each participant. To give the initial understanding of the capabilities of voice assistants in hospitality, it was decided to show consumers a 1:38 min video about *Alexa for Hospitality* as a part of experiment [46]. Zoom provided the opportunity to talk directly with respondents and clarify issues. All interviews were digitally recorded for further transcription by the researchers. Email offered more time flexibility for respondents and an opportunity to get information at their convenience. Email response simplified transcribing, saved time and helped to overcome time zone differences with some participants, although, it eliminated non-verbal language and emotional factors. Semantic thematic analysis was applied to this study focusing on meanings [4]. Coding was facilitated by the *QDA Miner 5* software, that enabled to identify patterns in content using themes, subthemes and categories [4].

## Results

**Fig. 1.**
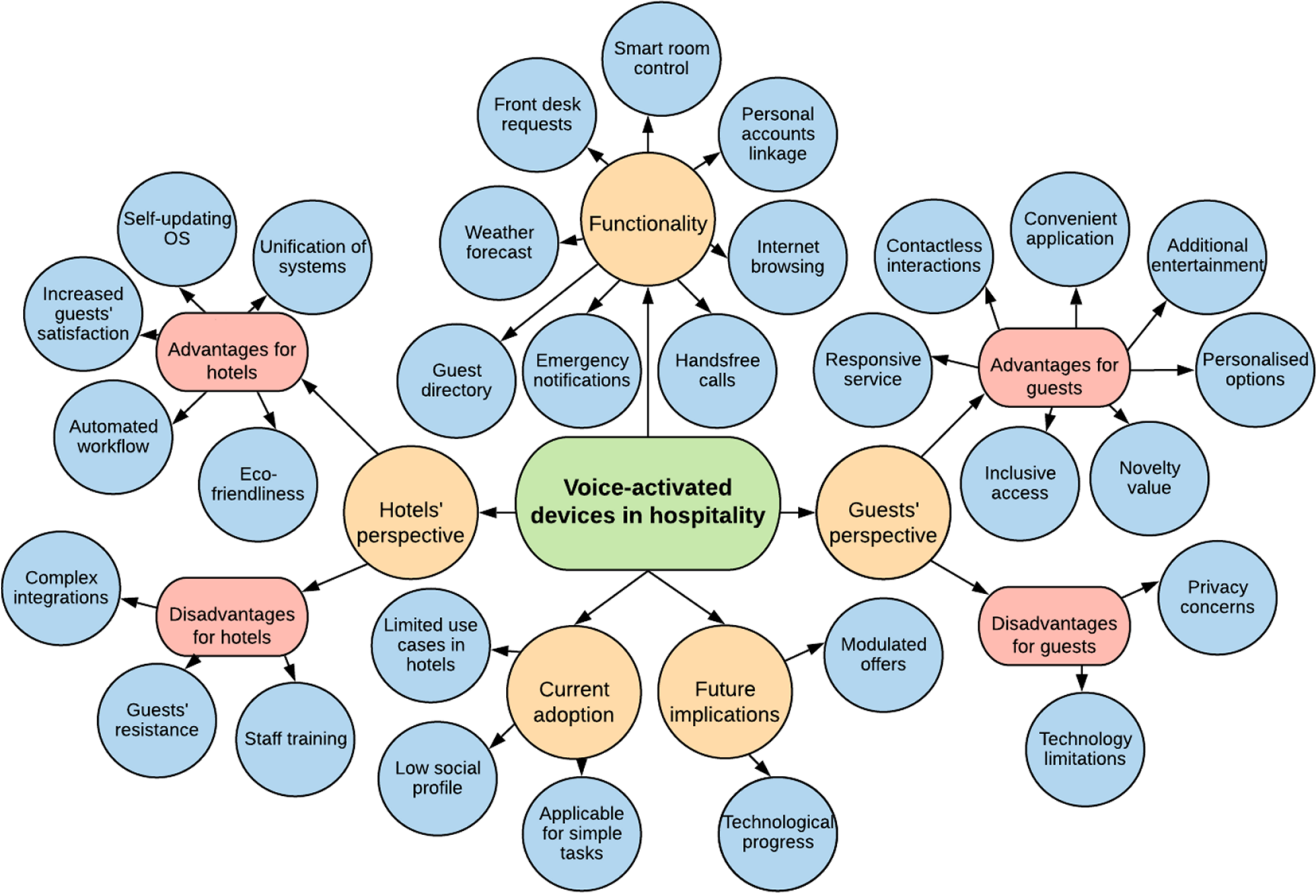
Advantages and disadvantages of hotel voice-based AI digital assistants

Responses provided a very rich overview of the hotel voice-assistants adoption. Guests expressed their expectations and the functionality required. They also shared their opinion on using this technology. Technology solution providers explained the technical capabilities and the core benefits. The map of themes and subthemes of all conducted interviews is presented in Fig. 1.

### Current Level of Adoption of In-room Voice Assistants by Hotels

Literature states that the adoption of in-room AI-empowered voice-based digital assistants for enhancing hotel services and experiences remains This study explores the reasons behind the current low level of adoption, such as: limited number of use cases to learn from in the industry and low awareness of the technology and its capabilities in society. This study also shows that experienced consumers believe that voice-based assistants can only fulfil basic tasks.

*“So, if I turn up to a hotel room… my first thought wouldn’t be to walk in and just start barking orders at an electronic device.”*

*“I don’t trust the computers enough to solve complicated problems. Because sometimes at the desk, you can have extraordinary situations where you would need extraordinary thinking rather than just the algorithmic thinking…”*

Providing guests with information on the functionality of voice assistants and issuing clear instructions on how to use them helps eliminate the barriers for those consumers who have never used smart speakers. Extra guidance from hotels provides more tech-savvy guests with deeper understanding of the usability and utility of voice devices.

*“Wynn [Wynn Resorts] has done a nice job with putting little placards in the room to sort of tell them [guests] what they [voice assistants] are able to do.”*

### Functionality of Voice-Based Digital Assistants in Hospitality

The functionality of Voice Assistants in hospitality was examined and summarised in Table 1. The results of the interviews acknowledged the main tasks frequently mentioned in secondary data sources, e.g. front desk requests, smart room control, Internet browsing, the weather forecast, hands-free calls, and guest directory. Yet, interviewees suggested additional functions and offered potential areas for development. For example, efficient feedback and complaint handling; Check out processes; facilities booking Maintenance, transfer, and weak-up requests, as well as controlling the physical infrastructure of the room including, temperature, lights; curtains and media devices. The possibility of smart speakers to serve as a deliverer of emergency notifications. This feature can be very useful if set properly.

**Table 1.** Functionality of voice assistants in hospitality

Themes	Subthemes	Categories
*Functionality*	Front Desk Requests	Room Service
		Feedback and Complaints Housekeeping Check out Facilities Booking Maintenance Transfer Wakeup Call
	Smart Room Control	Temperature
		Lights Curtains Media Devices
	Emergency Notifications	
	Weather Forecast	
	Guest Directory	
	Handsfree Calls	
	Personal Accounts Linkage	Calendar
		Shopping Lists

*“If someone breaks into your room it’s much faster to say with the voice command that you need help than to actually try to reach your phone.”*

Nevertheless, this function must be adjusted and tested before employing, as one participant contradicted the usability of digital voice devices for emergency situations, reasoning that technological limitations can prevent this feature from working properly.

*“You can say ‘Alexa, there is an emergency’, and the person that is breaking in can say ‘Alexa stop’ and Alexa will stop… I think in Alexa you have a setting to restrict it to your voice, but I wouldn’t see it happening in a hotel.”*

According to participants’ insights, the linkage of personal accounts and profile portability, can bring an additional layer of personalisation. This would be an auxiliary stimulus for some consumers to use voice assistants more willingly in the context of their stay in hotels. However, there are security and privacy concerns, as this is often been associated with data breaches in broadcast media.

### Hotels’ Perspectives on Digital Voice Assistants

The advantages and disadvantages which accompany voice-enabled interactions in hotels were analysed from hotels’ perspectives and presented in Table 2.

**Table 2.** Hotels’ perspectives on the usage of voice assistants in hospitality

Themes	Subthemes	Categories
*Advantages for Hotels*	Automated Workflow	Staff Offload
		Seamless Routing of Tasks Reduced Operational Cost
	Increased Guests’ Satisfaction	
	Unification of Systems	
	Self-updating OS Eco-friendliness	
*Disadvantages for Hotels*	Guests’ Resistance	Age/Demographics Preferences
		Importance of Human Service
		Avoidance out of Habit
	Complex Integrations	
	Staff Training	

#### Advantages for Hotels.

In-room voice-activated devices often reduce labour cost and provide coverage around the clock. They can reduce service friction and allow staff to spend more time on enhancing guests’ experiences. Given that in-room voice-based digital assistants are part of Internet of Things (IoT) systems they are integrated with hotel amenities and back office [8, 10, 30]. That empowers staff to stay constantly informed on guests’ requests and experiences, respond rapidly, pass messages on to relevant department efficiently when service requests or complains are identified, using contextual and real-time information [6, 9]. Most technology providers mentioned automation of processes as the main advantage of voice assistants for hotels. The benefits that can be associated with the automation of workflows were determined by participants. These points fully match with those outlined in the literature.

*“We saw a huge uptick in the satisfaction of the front desk team, their ability to provide a better service because they weren’t answering phones…”*

Operational efficiency, that is usually mentioned in literature with regards to automated workflows, has also a strong influence on guests’ satisfaction [24].

*“With an automated system everyone in the hotel can ask for an extra pillow at the same time and that can then go into a workflow. So, they all know that their request is being heard and that’s a great customer experience.”*

Respondents also revealed that voice assistants can offer more eco-friendly operations, cost-effective maintenance due to self-updating OS, and the unification of interfaces.

*“Ability to add incremental features and capability without any ‘forklift upgrades’ as the smart speaker capabilities improve.”*

*“The technology behind the voice activation facilitates a parallel automation system that can reduce the consumption of energy.”*

*“Unifying a variety of systems which originally had different modes on interfaces, into a singular mode, voice.”*

#### Disadvantages for Hotels.

Though the cost of voice speakers is low, to function efficiently, they must be interoperable and interconnected with other hotel systems [10]. Therefore, before benefiting from cost-saving mechanisms, hoteliers must invest in their IoT network. The complexity, challenge and cost of integration was endorsed by all technology providers during their interviews.

*“The challenge that you have with a hotel is that every single hotel is very unique… So, integrating with all the management, food and beverage, billing software, that’s usually different on a per hotel basis.”*

While reviewing disadvantages of voice-activated devices, it was crucial to analyse some consumers’ resistance to technology. The predominant number of participants shared hoteliers’ concerns regarding guests’ resistance to using voice assistants in their rooms, connecting this barrier primarily with habits and demographics.

*“People are creatures of habit. So, if you are used to setting alarm every day on your phone, maybe you wouldn’t see the point of setting it on the Google/Alexa.”*

*“Older people might be somewhat reticent to use this kind of device finding it a bit odd to talk to a computer.”*

There is still a belief that no technology can potentially replace human service delivery. Such statements were made by the minority of participants, as all the interviewed consumers had used voice assistants, whilst technology providers clearly had a vested interest in voice technology expanding its market share in the hospitality domain.

*“Some people, they prefer a human touch. You know, they are preparing for a service to be delivered. They believe that part of what they’re paying for is as a human service.”*

### Guests’ Attitudes to In-room Voice Assistants in Hotels

Guests’ ideas regarding the benefits and limitations of speech-based interactions in hotels were investigated in detail and illustrated in Table 3.

**Table 3.** Guests’ perspectives on the usage of voice assistants in hotels

Themes	Subthemes	Categories
*Advantages for Guests*	Responsive Service	
	Convenient Application	Time-saving
		Human-like Response
		Single Point of Access
		Similar to Personal Voice Devices
	Additional Attraction	
	Novelty Value	
	Contactless Interactions	Hygienic
		Unbiased
	Inclusive Access	
	Personalised Options	
*Disadvantages for Guests*	Technology Limitations	Language and Accent Recognition
		Semantic Analysis
		Multiple Device Conflict
		Dependence on Wi-Fi
	Privacy Concerns	Personal Data
		Industrial Espionage
		3rd Party Scams

#### Advantages for Guests.

Voice assistants are available 24/7 and transfer requests to relevant hotel services immediately. This level of responsiveness empowers hotels to meet guest demands for instant gratification [7], as mentioned by participants.

*“When you arrive in your room, you probably want to set your environment out for yourself, order a late check out, get another pillow, order your dinner. And that can be a long kind of half*-*hour process if you are trying to phone and get all these bits of information from the hotel. If you can just know that there is an automated agent to get these tasks done for you, then you are going to have a better stay.”*

*“Among advantages for guests: Intuitive Interactions, Commands, Responses as if speaking with a human”*

In addition to bringing ease to digital interactions with hands-free features, time-saving opportunities, and the advantage of having a human-like communication, there is also a key aspect of meeting guests’ technological habits.

*“If you have the same system of assistant in your home, it’s pretty convenient for you because you know what you can ask.”*

The sense of novelty and the additional level of entertainment, which new technology often provides, has also been defined within this study as a benefit for guests.

*“At this moment, it’s also kind of a novelty… You don’t necessarily need it, like you can turn on the light by yourself, right? But it is much fancier and unusual to do it with a machine…”*

Consumers who took part in this research admitted that using voice assistants in hotel rooms can potentially enhance their experiences by providing inclusive access, personalised options, and contactless interactions. The findings suggest that hoteliers should not fully rely on the COVID-19 pandemic-related trend for minimising face-to-face service delivery but to fully explore the opportunities of this technology for customer service.

Among the reasons for preferring contactless interactions named during the interviews were: hygienic interactions (regardless of the pandemic) and unbiased characteristics of voice assistants. As discovered during the interviews, people who prefer face-to-face service delivery are not likely to change their minds due to new COVID related health regulations. The same is true for those who prefer being served by computers.

*“You don’t have to touch things, like light switches that are usually very dirty…”*

*“Lowering the barrier to making requests, not having to feel that burden of asking for something.”*

#### Disadvantages for Guests.

Despite all the developments of NLP, existing voice-based assistants still struggle when dealing with accents and foreign languages [14]. This has been broadly disclosed in papers as well as by the majority of interviewees who underlined the importance of this drawback, since many hotel guests arrive from abroad.

*“So, if you speak with a French accent or if you’re referring to the French author, and you pronounce the French name in a French way, the machine doesn’t get it because it speaks English to you.”*

While reviewing technical limitations of voice assistants and the occurrences that lead to malfunctions, participants identified “multiple device conflict” and “dependence on Wi-Fi” as reasons that significantly influence interactions with voice speakers. Scholars [36, 40] agree that privacy is still a major concern associated with voice assistants. It has unsurprisingly been the most frequently named disadvantage of voice assistants, reflected in this study by almost all participants, primarily from a personal data point of view. Nonetheless, some participants were particularly wary of industrial espionage that had not been discussed in literature.

*“It’s one thing having it in your own home, and a lot of people don’t necessarily want that, but you have some semblance of an idea that you control it because it’s your account. So how does that switch over work when you are in a hotel? Is the data actually being harvested by the Hilton or whoever for use in there?”*

*“If I am running a meeting from my hotel room and technically, we sign NDA [non*-*disclosure agreement] with our clients. And… Amazon may listen to everything we discuss. And technically, it may be a part of business that competes with Amazon or maybe a start*-*up that may be acquired by Amazon.”*

However, this research proved that more tech-savvy consumers tend to be less concerned about privacy when using voice assistants.

*“I personally don’t have concerns about privacy, but lots of people have. I stand for open information flow… IT people know that Google, Apple have lots of information about us even without hotel assistants.”*

### Future Implications of Voice-Based Digital Assistants in Hospitality

**Fig. 2.**
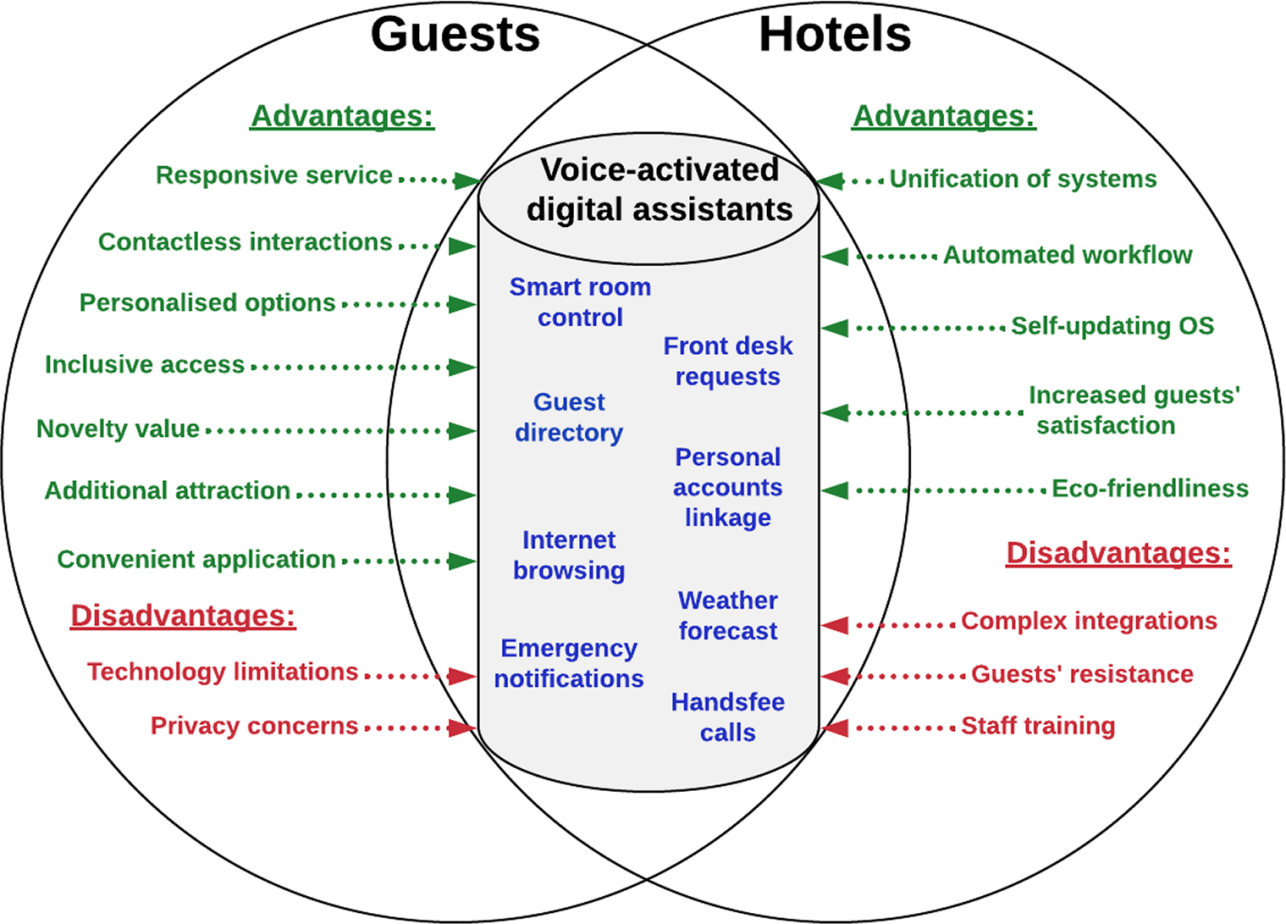
Conceptualisation of speech-based interactions between hotels and guests

The growing connectivity of devices and the Internet of Everything [6, 8] justifies the inevitable technological progress in hotels [10, 30]. The only question would be what role in this progress voice assistants will play. Figure 2 presents the conceptualisation of speech-based interactions between hotels and guests. Digital voice assistants’ producers aim to embed their technology into as many devices as possible, allowing brands to easily create voice-compatible products, e.g. TVs, headphones, smart plugs, bulbs, locks, security cameras, soundbars, watches and tooth brushes. This enables major technology manufacturers to have a wider pool of sources for their AI software to collect data, label it and learn from it. Consumers are getting used to being hyper-connected via voice as an interface. They are not only using standalone or smartphone in-built assistants, but also through almost every device they use throughout the day. This is likely to impact hotels in future, when in-room smart speakers become irrelevant as guests will be able to access voice assistants from any digital device in their rooms. Yet, the ability of in-room voice-activated devices to understand and speak different languages and engage in different accents [39] was identified as the defining feature for their future applicability. Some participants shared the statements that a wider adoption of the technology will have a differentiated or modulated approach [25]. This effectively mean that it would be useful to have voice assistants as an option for guests, rather than a default setting, provided that the devices can be managed centrally.

## Conclusions: The Future of Voice Assistants in Hotels

Despite their limited adoption by hotels, it is clear from this research that the advantages of using voice assistants in hotels outweigh the disadvantages for both hotels and guests. Guests’ expectations of their functionality acknowledge the core tasks frequently mentioned in secondary data sources and demonstrate their use. In addition, technology solution providers illustrate the emerging technical capabilities of these devices. Yet, this research unveiled some points which had not been reflected in existing literature. For example, the possibility of smart speakers in hotels to serve as a deliverer of emergency notifications or bring an additional layer of personalisation through profile portability with the linkage of personal accounts.

The findings illustrate that voice-based human-computer interactions bring a range of benefits and voice assistants will be widely deployed in the future. To fully benefit from their capabilities, hotels will need to ensure the interoperability and unification of their systems within the IoT infostructure. Technology integrations are often complex and costly to set up but provide significant benefits. Guest appreciate the prospective benefits but are concerned with privacy and usability, although tech-savvy consumers are less concerned about privacy when using voice assistants. With all participants believing in technological progress, the findings indicated the direction for the future development of voice technology in hospitality towards multilingualism and modulated offers which can ultimately ensure the overall wider reach of the technology in the hotel industry.
